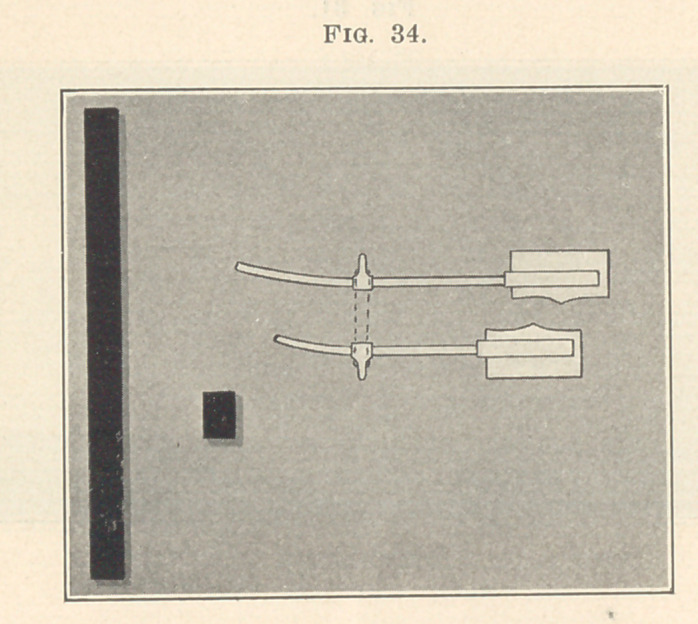# A Consideration of the Occlusal Balance

**Published:** 1905-12

**Authors:** Lawrence W. Baker

**Affiliations:** Boston, Mass.


### A CONSIDERATION OF THE OCCLUSAL BALANCE.[1]

BY LAWRENCE W. BAKER, BOSTON, MASS.

ORTHODONTIA is a science broad and interesting. The illustrations which I wish to show you have been selected and arranged to prove the truth of this statement. The subject is so broad that I can only bring out a few fundamental principles on which the science is based; but by exhibiting the results which I have obtained by working on these principles I hope to hold your attention this evening.

In dealing with the subject I shall depend largely upon the stereopticon; in fact the paper will consist principally in the description of slides which have been selected, grouped, and arranged to bring out a few fundamental principles underlying this work.

The first group of illustrations deals with the application of force and Fig. 1 shows the old well-known expansion arch appliance in its simplest form adjusted to a typical case for treatment.

The true progress of orthodontia,—and this is one of the very few unqualified statements I shall make this evening,—depends upon this appliance and its modifications. The single fact that it is the only appliance in which the necessary forces are reduced to their lowest terms is sufficient proof of the truth of the statement.

The appliance as we see it represents the results of centuries of work in simplifying. Even as far back as Fauchard's time these same principles were well-known and practiced. Each true step in advance from that time to this was a more direct and simple method in the application of force, until the appliance now has reached perfection. The appliance is perfect because, as I say, the forces are reduced to their lowest terms; that is, to three distinct and positive forces: the spring of the arch-wire, the ligature force, and the screw force. All these act and react in harmony upon the arch through the anchorage and arch-wire. These three primary forces can be applied in many combinations; in fact, they are capable of as wonderful and innumerable combinations as those of the ever-changing kaleidoscope.

---

[1] Read before the New York Institute of Stomatology, Nov. 4, 1905.

Fig. 1.

Fig. 2.

Fig. 3.

Fig. 4.

Fig. 5.

Fig. 6.

Fig. 7.

Fig. 8.

Fig. 9.

Fig. 10.

Fig. 11.

Fig. 12.

Fig. 13.

FIG. 14.

Fig. 15.

Fig. 16.

Fig. 17.

Fig. 18.

Fig. 19.

FIG. 20.

FIG 21.

Fig. 22.

Fig. 23.

Fig. 24.

Fig. 25.

FIG. 26.

FIG. 27.

FIG. 28.

FIG. 29.

FIG. 30.

FIG. 31.

Fig. 32.

Fig. 33.

Fig. 34.

Now let us consider one or two typical examples of tooth movement accomplished with this appliance.

Fig. 2 shows a case that is a severe test for this or any appliance, a badly contracted, pinched, and distorted arch caused by adenoids and mouth breathing. Fig. 2*b* shows the result of the work of this appliance in its simplest form. Note the measured expansion and general enlargement of the arch, allowing room for the partially erupted cuspids to be brought into place; note also the rotation of the incisors.

Fig. 3*a* is another example of a condition where the tongue was noticeably cramped; Fig. 3*b* shows a new arch, chiselled out, we might say, by the appliance. Again note the expansion as shown by the calipers. As the arch was expanded the alveolar process followed the teeth, building in the new bony vault. So you see the law that the alveolar process is subservient to the positions and uses of the teeth is as true of applied forces as of natural forces.

The next illustration (Fig. 4) shows normal occlusion. It is the model from which I work and it is regarded as the model by all who consider orthodontia a true science. A wonderful piece of nature's work it is, wonderful from the standpoint of art and proportion, from the standpoint of hygiene and utility. However, it is not for these reasons that I spent an entire Sunday morning in obtaining this negative. The reason why I want you to see every visible line, curve, and contour is this: when the teeth are in their normal positions they are in their most retentive state, in their most retentive positions; they are in a state of equilibrium, occlusal equilibrium. There is an exact correlation of the occlusal forces which can be produced in no other conceivable arrangement of the occlusal planes. It is for this simple reason that true orthodontists consider normal occlusion their model. Without stopping to consider the law of this balance,—how and why each and every cusp is so shaped and arranged, how the muscular pressure of the lips and tongue aids in maintaining it,—I shall deduce one simple rule, a rule with few exceptions: A corrected case of orthodontia is a success in proportion to the approach or establishment of the occlusal balance.

From Fig. 4 it is also possible to see the classification which has been made by Dr. Edward H. Angle and which is perhaps the greatest step that has been taken towards placing orthodontia among the true sciences. Using normal occlusion as a working basis, with the first permanent molars as the primary landmarks, Dr. Angle has given us a most adequate classification of the various forms of malocclusion. It is the classification that I have adopted in the grouping of the cases that I am about to show you. No doubt you are all familiar with it, so I shall not consider it minutely. Roughly it is this: Class 1. Irregularities in which the first permanent molars are normal mesio-distally. Class II. Irregularities in which there is a distal displacement of one or both the lower first molars. Class III. Irregularities in which there is a mesial displacement of one or both lower landmarks.

With this introduction we will now consider the cases.

Case I, Fig. 5, shows a very ordinary type of the first group. It belongs to this group because of the normal position of the molar landmarks. The antro-buccal cusp of the lower first molar one plane in advance of the upper ones as indicated by the pencil lines. The eye falls at once upon the tusk-like appearance of the upper cuspids, exaggerated perhaps by the sunken and depressed positions of the laterals. You all know that if this case were left uncorrected, the laterals, from their instanding position, would suffer sadly from dental caries, and the cutting edges would become badly worn.

Fig. 6 shows the result of the attempt at approaching our model normal occlusion. There is a marked improvement in appearance, an increase in utility, and the danger from dental caries is greatly minimized. All these result from the fact that the occlusal balance has been established.

Case II (Fig. 7) belongs in Class I because the molar landmarks are normal; otherwise it differs widely from Case I. It is a much more difficult case than the first, nearly every tooth being in malocclusion. Nature has been so interfered with that her laws of self-cleansing have been entirely overthrown. Look ahead some years and imagine the probable condition.

Fig. 8 shows the result. I am sure the occlusal balance was reached, for after I had done my best the case steadily improved and since the removal of the retainers has kept on improving. Nor was the improvement confined to the occlusion: the facial lines have improved in proportion. A sad fact regarding this case,—and the same is true of the majority of cases,—is that the deformity could have been largely prevented. Fig. 9 shows the cause, the injudicious handling of the temporary teeth. The remains of the deciduous teeth have deflected the incoming permanent teeth until these occlusal planes were forced out of the normal relation; and when once beyond the normal these same forces, instead of working for the balance, work directly against it. The result is an ever-increasing deformity.

Case III. This case is interesting because it illustrates another force of the dental arches that we have not yet considered, the mutual contact force.

Fig. 10 shows the conditions before treatment. The case belongs in the first group, since the molar landmarks on both sides are normal. Note the missing cuspid and its partly obliterated space. Above the space upon the gum is the characteristic swelling indicating the position of this impacted tooth.

The arches were enlarged to make room for the missing member, the space was retained, and the patient was dismissed for the summer. Fig. 11 reveals the condition of affairs when the boy reported for treatment this fall. The cuspid is in place and the lower retainer is doing its work admirably. My plan now is to complete the slight adjusting of the occlusal planes necessary to obtain the perfect occlusal balance.

Now comes the interesting part of the case. Fig. 12 shows the individual views of the arches before treatment. Note in the lower arch the contraction through the anterior region, a corresponding deformity which is nature's way of making up for the missing cuspid in the upper arch. It is a never-failing rule that when one arch is deformed there is invariably a corresponding deformity of the same kind in the opposing arch.

To return to the case. What I especially want to call your attention to is the width of the arch, as shown by the calipers. Compare it with the relative width of the arch after treatment in the next view.

In Fig. 13 this expansion is clearly shown. The enlargement of the arch is not remarkable in itself being about one half the width of a bicuspid; but it was maintained when once established by the natural forces and not by applied forces. The cuspid, wedging it way down into place, held the enlargement, and it was further aided by the lower arch, which, when remodeled, acted normally through occlusion. This case is presented not with the intention of giving you the idea that it is a difficult one, but as an illustration of the reward for assisting nature.

Having considered these three cases, which are fair types of the first group, we will now pass on to the second group, which to me is the most fascinating and interesting of all classes of cases that the orthodontist is called upon to treat. I doubt if within the entire province of dentistry there is a branch which is capable of such gratifying results. These cases are fascinating, interesting, and gratifying simply because of their great benefit to humanity. They benefit humanity more than any class of operations that we as dentists are called upon for treatment.

Not only do these deformities cause the greatest impairment to the functions of the teeth, jaws, and vocal organs, but they cause a most unsightly and distressing facial disfigurement.

The results of these deformities are far reaching: they go deeper than mere facial disfigurements, they penetrate even to the highest nerve centres, and I believe that there is a definite psychological effect produced by the correction of these deformities.

We are now to consider a series of faces illustrating these facts, and I want your opinion as to whether or not there is a psychological change produced in the treatment of these cases. Fig. 14 shows a subject at our clinic at the Harvard Dental School.[2]

The illustration shows a boy fifteen years of age. In the upper left-hand picture the full face is shown before treatment. It is a typical illustration of the expression of a sufferer from distal occlusion in its simplest form. Note the vacant stare. Below is the profile taken at the same time. Again see the weak expression of the mouth caused by the recession of the chin, and exaggerated by the protruding incisors. It was not possible

---

[2] It is through the courtesy of Professor E. H. Smith that I have the pleasure of presenting this particular case to you. I must digress just a moment to say one word regarding this series of photographs. The negatives were not in any way re-touched, the prints were not altered, nor were the patients posed so as to exaggerate either the deformity or the result. The patients were simply instructed to take a natural position.

for the boy to cover these teeth with his lips except by conscious effort.

On the right we see the results of treatment,—a bright, intelligent face. Below is shown a good profile caused by the restoration of the occlusal balance. The result of which is the maximum amount of masticating surface, normal respiration, normal tongue room, normal muscular pressure of lips and tongue; and above all and with all, normal facial expression. Also notice that the boy has spruced up a bit: a flower appears in his buttonhole, his collar and necktie show signs of more tidiness, his hair a little more care in arrangement; in all, a little more self respect. Is this change a coincidence or is it a result?

Figs. 15 and 16 have been lent by Dr. H. L. Howe and fine examples they are of the improvement to the facial lines.

Now, gentlemen, I want to consider with you regarding these faces. I want to get at the truth of the matter. Is there a relation between these disfigurements and the brain, or is this a mere optical illusion produced in the correction of these cases; or is the improvement due to the establishment of normal respiration, or is it due to the general effect of the full power of mastication, or is it the combined results of these factors that give the boy his manly carriage and set-up? In short I want your opinion as to whether or not a psychological change has taken place.

Figs. 16 and 17 are other examples illustrating this same point.

In each and all of these children whose faces we have just considered, not one tooth was lost through neglect, or removed for correction. The laws of occlusion were sacredly regarded, with gratifying results to the facial lines, as we have just seen.

The method used in the treatment of these cases is interesting because it is the final step that placed orthodontia among the true sciences. Like all other great advances, it is so simple that its origin is obscure. It is impossible to say who was the first to use it. However, I am pleased to state that my father, as you all know, was one of the pioneers in introducing this most valuable method. He was the first, as far as I am able to learn, to use it with marked success, the first to obtain the occlusal balance. Because of the far-reaching influence of this method in his hands, it bears the name of Baker anchorage. We must not, however, forget that Dr. Case of Chicago worked along the same lines as my father, although each was entirely independent of the other.

Fig. 18 is a diagram, original with Dr. Angle, illustrating the underlying principles of the Baker anchorage which is called by some the intermaxillary elastics. In the upper figure we see the appearance of the two arches in distal occlusion, with the appliances in position. When the lower arch is in distal occlusion, you remember it occludes one occlusal plane back of its normal place, as is clearly seen here. In the figure below, the elastic, indicated by the dotted line. is looped over the distal end of the tube of the lower anchor band, passed forward and engages in the hook on the upper arch wire. At one side are the elastics which are especially prepared for this particular use. You readily see that this is just the necessary force to correct this class of deformity, pulling forward on the lower arch and backward on the upper.

By the skilful handling of this elastic force, in combination with our three primary forces, it is possible to obtain the pleasing facial results that we have already considered, and the occlusal results that we are now to consider.

Case IV, Fig. 19, is the first case of the second group, that in which the Baker anchorage plays such an important part. This case is unmistakably different from the former cases. When we study the occlusal relations, we find that our landmarks are out of their normal position. The lower arch is one plane back of its normal place. It is in distal occlusion. The entire dental apparatus, we might say, is dislocated; the lower jaw is dislocated distally and with it naturally the lower part of the face is distorted, as you have already seen from the photographs.

This dislocation was formerly treated by mutilation. The upper arch was mutilated by extraction, two or more bicuspids being sacrificed to compensate for the malposition of the lower arch. This was before the time of the intermaxillary elastics. But now, by the aid of these elastics, the occlusal balance is made possible.

Fig. 20 shows the result of the use of the Baker anchorage,—the ever-sought occlusal balance.

Sometimes a fear is expressed that the lower jaw will fall back to its old position. It is impossible for this to occur because the upper arch has been remoulded, and widened, and the old position of the lower jaw has been destroyed. If the lower jaw falls back, the cusps will strike point to point. The patient will not tolerate this condition, but will slide the jaw forward into its normal place, where the cusps will interlock.

Figs. 21 and 22 are the photographic record of the results of the occlusal changes upon this child's facial expression.

In Fig. 21 (left) note the decided lack of balance between upper and lower portions of the face. The part above the upper lip is well-proportioned and strong, the lower part is weak and receding. The result is an inharmonious whole.

On the other side we see both views after one month's work. The lower jaw has just been brought forward, producing a great improvement in the facial contour but not so great an improvement in the expression, for there is a more or less set and constrained appearance.

Fig. 22, (lower left view,) which was taken one month later than the preceding, shows that the expression is adapting itself to the facial contour.

The next view taken just eight months after the last one. You see it is a most pleasing expression.

(Fig. 17). The comparison again. The time elapsed between these two pictures is just ten months and eighteen days. During this time the dental arches, we may say, have been remoulded, and the lower jaw rearticulated. The effect of these changes upon the facial expression you can judge for yourselves.

Case V, Fig. 23, shows a new kind of case, distal occlusion on only one side. This deformity Dr. Angle cleverly places as a subdivision of the second group.

This sub-class is considered by all difficult to treat because it is hard to make the arches normal. Each lateral half of each arch is treated differently. The distal side is treated as a simple case of distal occlusion: the normal side, as a case of the first group. In this case as in all others, it was necessary to make sure that the occlusal planes fall within the normal influence and then to allow the case to settle.

Fig. 24 shows the case when it has settled enough so that the distal side will hold in its forward position. It will hold there because the old position into which the boy used to bite has been destroyed. When he now tries to bite back, the cusps strike point to point and he unconsciously slides forward to the present position where the cusps interlock properly. Thus the occlusal balance is secured.

Case VI, Fig. 25, is an unusual case of distal occlusion and one that has aroused much interest because the distal state of the lower arch was self-correcting. It is entirely different from either of the two preceding cases, because the incisors do not protrude, by retrude. Therefore it is classified as a case of distal occlusion with retruding incisors. In Fig. 26 which shows the individual aspect of the two arches before treatment, note that the upper incisors are badly crowded, and that the lower arch is perfectly normal. In Fig. 27 which presents the same views of the corrected casts, see that the upper arch has been corrected and that the lower arch has not been altered. In fact no appliance, elastic or other mechanical force of any description was used during the progress of the case. In Fig. 28 we see that the simple rotation of the superior incisors has allowed the lower jaw to come forward to its intended place. Formerly the lower arch was forced back into its distal position by the malposition of the superior incisors. When this condition was corrected the lower jaw simply slid forward to its proper place.

This case is introduced, not so much for its unusualness but rather because it illustrates an important detail in the use of the elastics. A great many in using this method apply so much elastic force that the anchorage is disturbed. This is not necessary. It is surprising what a slight elastic force will do provided it is intelligently applied. All that is necessary is to make the arches normal, and then to guide the lower jaw forward to the occlusal balance. It is amazing how soon nature appreciates the fact that she is being assisted, and how soon the new position becomes natural.

In the last case which I have to show you this evening, the deformity was great. In order to appreciate the changes that took place, it would be well to refer to the model normal occlusion shown in Fig. 4. It is seen there that the normal position for the cuspids, the secondary landmarks, is one in which the lower cuspid occupies the space between the upper cuspid and the lateral.

Fig. 29 shows the two side views before treatment. The marked deformity is due to a decided dsplacement of the two arches, principally of the lower. In an ordinary case of distal occlusion the point of the lower cuspid falls one space back of the normal position; in this case this point is one plane back of ordinary distal, or between the two bicuspids. Since in this extreme case the lower jaw recedes not one but two notches, the deformity might be termed double distal.

That the deformity caused a great impairment of the function of mastication, as well as a pronounced facial disfigurement, can be judged from studying this illustration. You may be sure that I must have had great confidence in the possibilities of the intermaxillary elastics, or I should have hesitated at the responsibility of undertaking this case.

When the boy came to me the lower six-year molars were beyond saving. So, at the proper time the bits of roots were removed, allowing the then erupting twelve-year molars to come forward into the spaces of the missing six-year molars.

In Fig. 30 the effects of the extreme distal position of the lower jaw upon its function are here clearly shown. The ten or twelve anterior teeth were made entirely usless for masticating purposes. It was possible to pass the forefinger between the two arches even though they were in close apposition.

In Fig. 31 are seen the two side views after treatment. The upper arch has been made normal, and the lower jaw has been brought forward to its intended position,—one notch, to simple distal, and another notch to normal,—greatly improving the facial contour, as well as increasing the utility of the entire dental apparatus.

In this case a great many would have considered extraction in the upper arch necessary. Probably the removal of two biscuspids would have been resorted to or possibly the entire bicuspid region would have been sacrificed. That is to say, another deformity would have been created equal to the already existing one. I firmly believe that by keeping the upper arch intact and bringing the receding jaw forward not only were the jaws given full power, but the facial lines were placed in much better balance. Instead of weakening the upper part of the face by moulding it to the weak receding lower jaw, the whole face was strengthened by bringing the chin forward to harmonize with the general facial contour.

Now here is another problem for you gentlemen to solve. In producing these changes what has taken place? Study the profile of the two casts. Is this change produced by the reciprocal movement of the teeth? Is it in the body of the jaw, or has there been a change in the temporo-maxillary articulation itself? My opinion is that there is something of all of these possible movements. I am sure that in the correction of these extreme cases,—to which we might almost apply the word "dislocation,"—there is a change produced in the joint itself. This theory is strengthened by the interesting case reported before this society by my father a few years ago which some of you may remember. It was a case of an excessive protruding lower jaw in which the changes that took place were accurately recorded by an indicator. In this case there was a measured movement in the joint of at least three eighths of an inch.

My confidence in this theory was still further strengthened by a most interesting morning spent with Dr. Cryer in his museum. He was decided in his opinion that there is a migration of the condyles in this case, and to confirm his belief he showed me specimen after specimen in which there was a migration of one or both joints. In some specimens the old articulations were completely obliterated; in others the path of the condyle could be definitely traced.

Fig. 32, another comparative view of the deformity, illustrates what we have already seen many times this evening:—first, that with the occlusal balance the jaws and teeth have been brought to their maximum usefulness; second, that this great increase is the very secret of the lower jaw remaining in its new position; and third, that this occlusal balance acts as a perfect and natural retainer.

Fig. 33 shows the palatal aspects of the upper arches before and after treatment. Note the great amount of expansion. The anterior teeth are held by the retaining device indicated; but no mechanical means whatever is employed to hold the extreme amount of expansion. The occlusal balance is the lock and the occlusal balance is the key.

One difficulty connected with the case proved very instructive. It was easy to make the arches normal, it was easy to bring the lower arch forward, but it was not easy to get the proper interlocking of the cusps. They would not settle because, with the lower arch in its extreme forward place, the two arches were moulded on different curves.

Fig. 34 shows the method of overcoming this difficulty. I applied a controllable and reciprocal elastic force between the two arches. I stretched a strong elastic over these spurs on the arch wire. This force, of course, is transmitted to the ligated teeth, and the arches are brought into closer contact. In fact, this is but a natural force,—the settling force,—magnified.

Perhaps the only novel idea about this simple method is the scheme for applying this elastic force,—the spurs fastened to the arch wires. Since it was difficult to get elastics short enough and strong enough for this purpose, I made use of the ordinary rubber wedging strips with suitable holes punched in them. The hole was put over one spur, and then the elastic was stretched to the other. Of course the strength of the elastic can be varied by the thickness of the rubber and the size of the hole. This simple method is introduced because it has since proved invaluable in other cases in obtaining that final and important settlement of the cusps.

Let me close with a few statements regarding the question that is at present agitating our profession,—extracting in orthodontia.

Personally I believe that the extremists of both the old and the new schools agree better than either will admit. Each side sees the situation from such entirely different points of view that to a casual observer they seem to disagree entirely; but, as I say, in reality they agree better than they will admit. As we become familiar with this wonderful appliance, the work of which I have but feebly presented, we find that the cases requiring extraction rapidly diminish in number. When we realize what the occlusal balance is, what its powers are, and how disastrous injudicious extracting is to this great piece of nature's work, we find that the cases requiring extraction are again greatly diminished, until such cases are few and far between.

However, once in a while and only once in a while, we find a case in which all the teeth are present, where extraction seems indicated; but even these cases are decreasing in number as our knowledge of the possibilities of this appliance increases.

Then there are cases in which the arches have been badly mutilated by the forceps, because of previous neglect. Of all cases these are the hardest and most discouraging to treat. Each of these cases is a puzzle in itself, but no law or rule can be applied to them; they are simply mutilated nature. In these cases, almost any method is admissible: extracting in some cases to balance lost planes, bridge work in others to supply lost planes; and in every case an attempt to establish a balance of some kind and remedy the facial distortion. As Dr. Smith says regarding these mutilated cases: "Obtain the best abnormal occlusion possible."

If I have been the means of causing any of you to look upon the occlusal balance with more respect I consider my efforts well rewarded; for the laws of occlusion are the very basis of the science of orthodontia: occlusion for utility, occlusion for retention, and occlusion for facial balance.

## Figures and Tables

**Fig. 1. f1:**
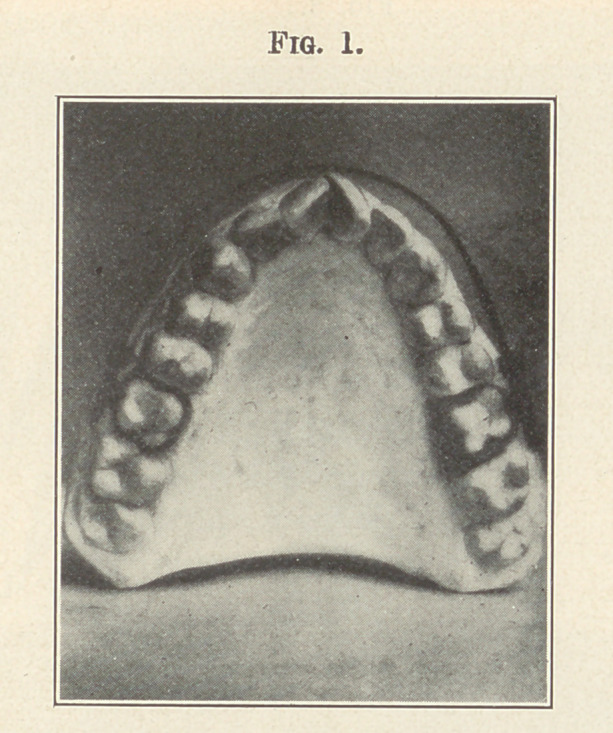


**Fig. 2. f2:**
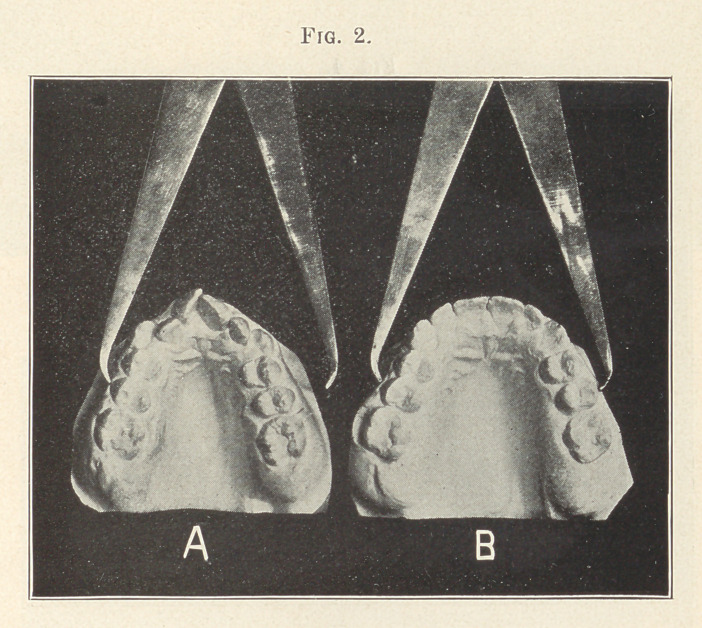


**Fig. 3. f3:**
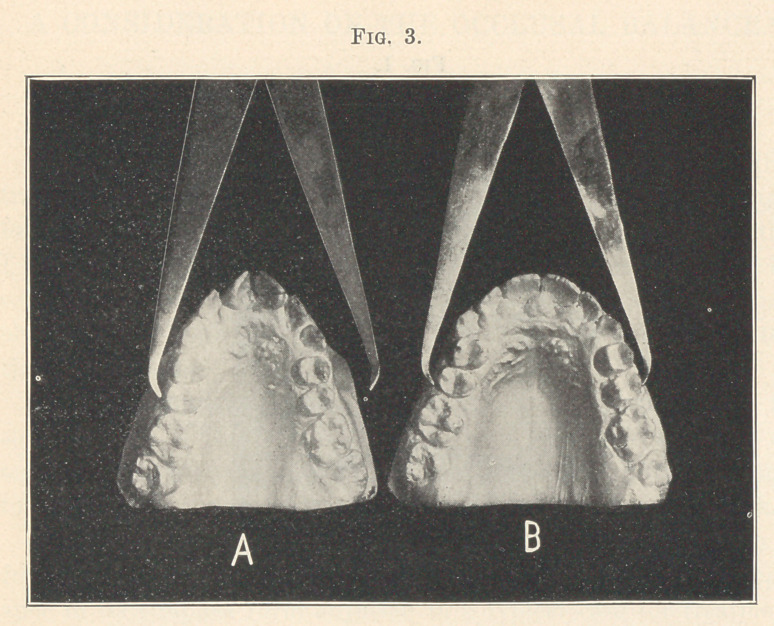


**Fig. 4. f4:**
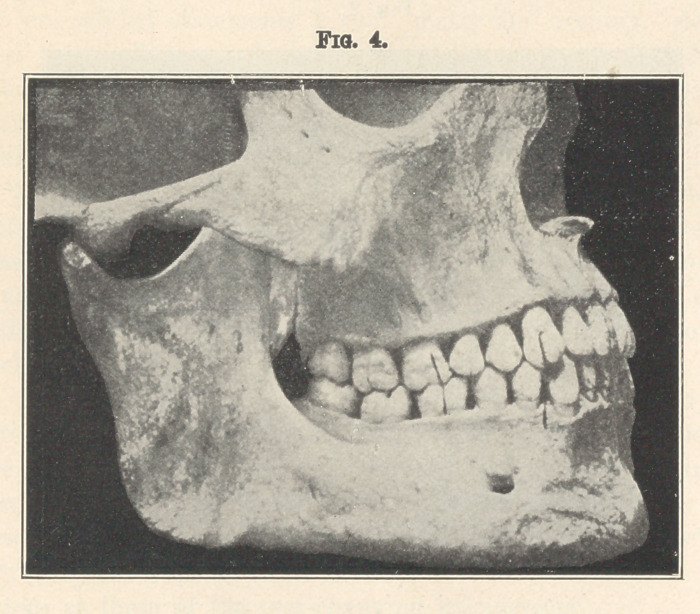


**Fig. 5. f5:**
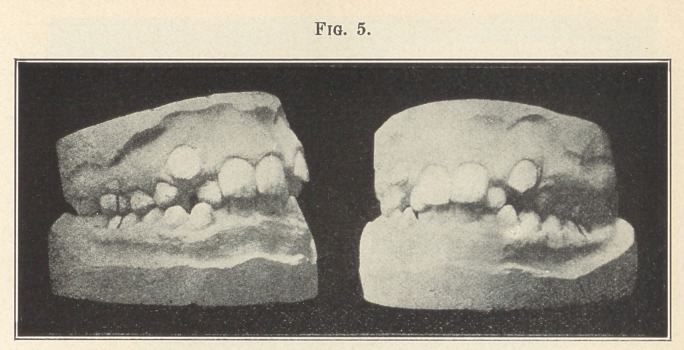


**Fig. 6. f6:**
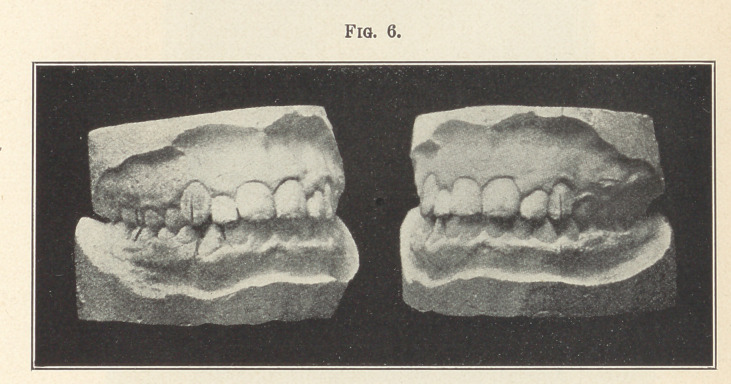


**Fig. 7. f7:**
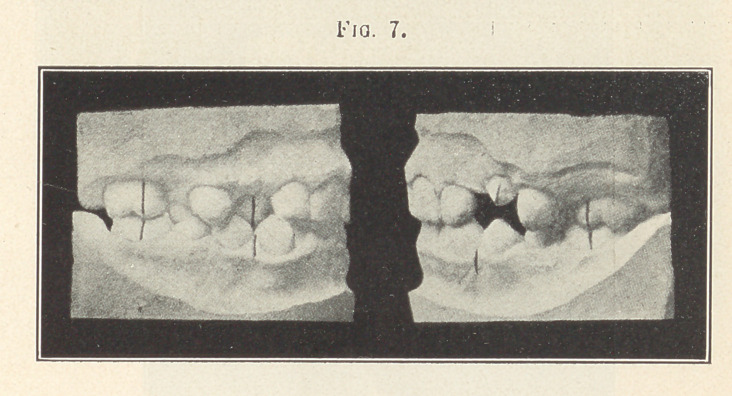


**Fig. 8. f8:**
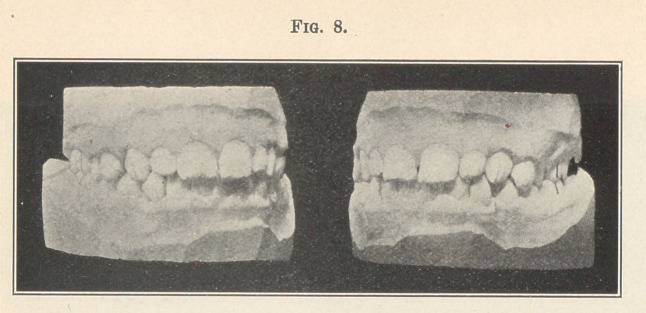


**Fig. 9. f9:**
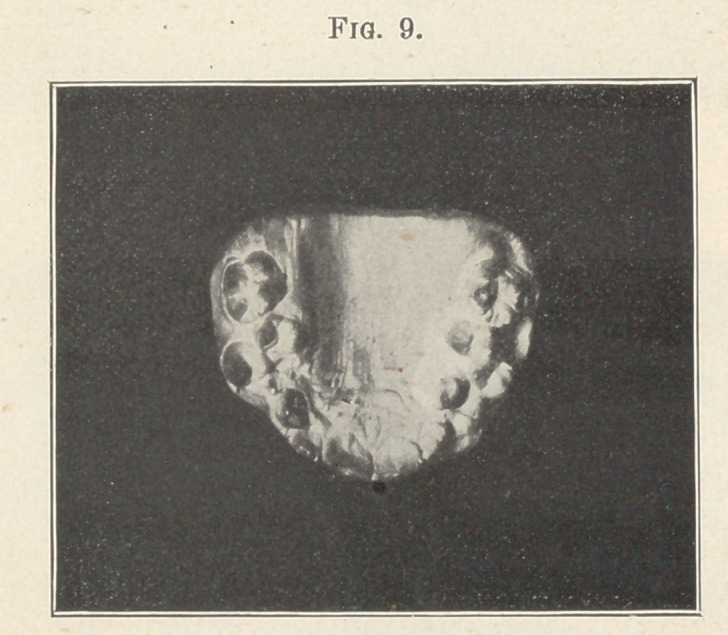


**Fig. 10. f10:**
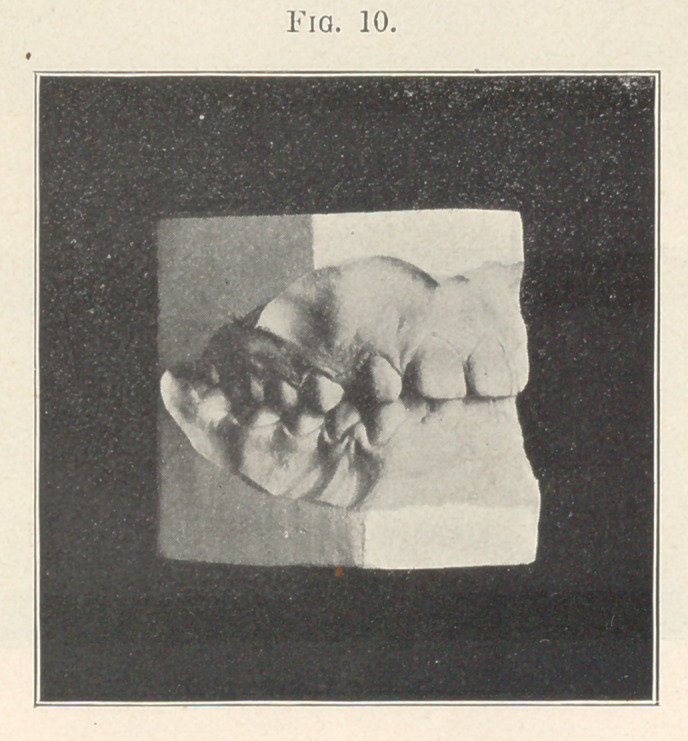


**Fig. 11. f11:**
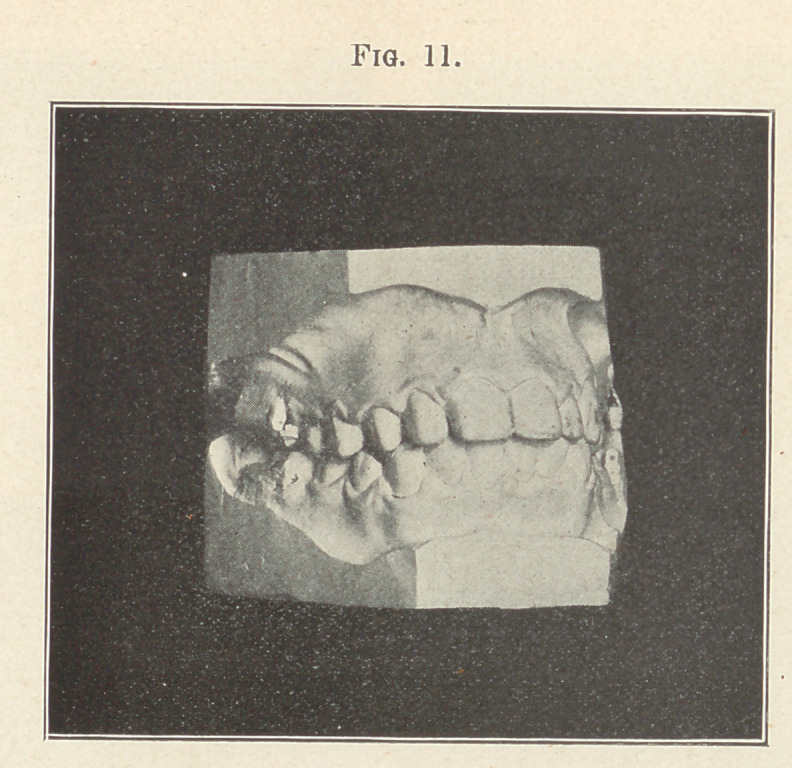


**Fig. 12. f12:**
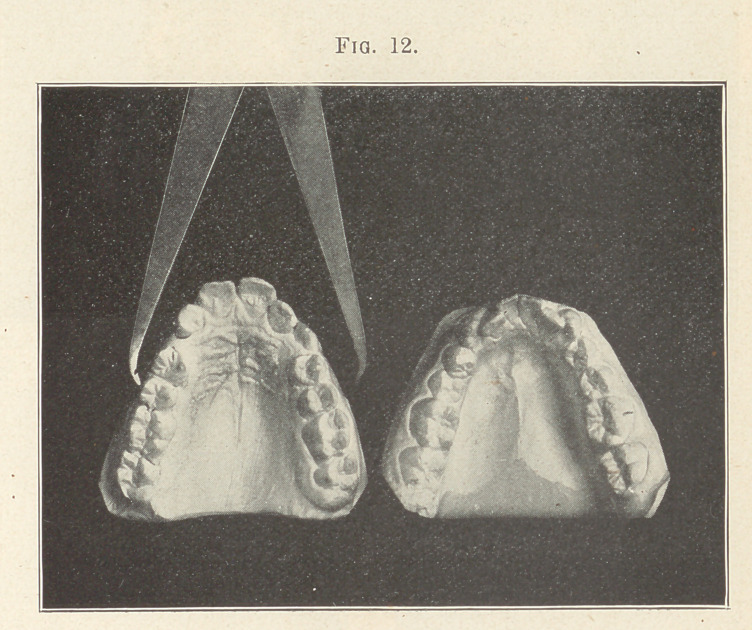


**Fig. 13. f13:**
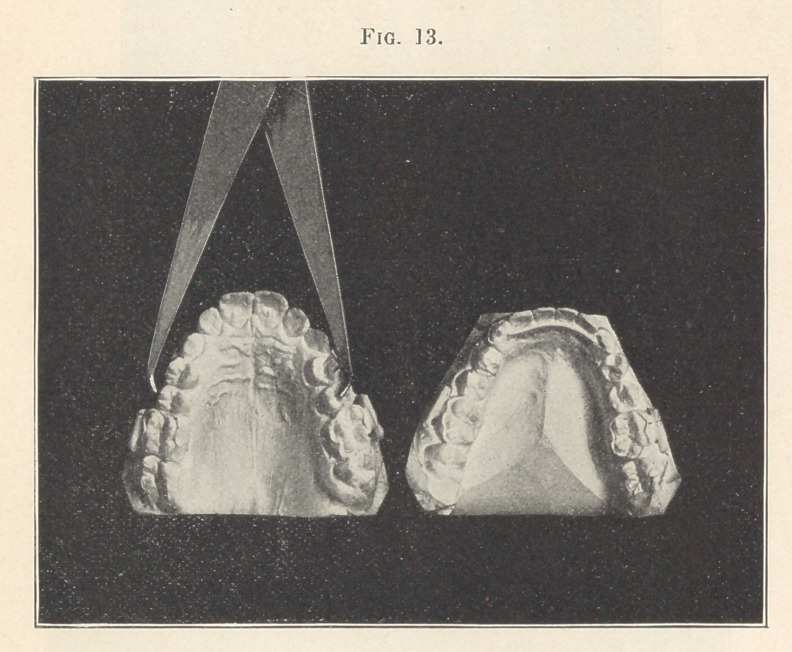


**Fig. 14. f14:**
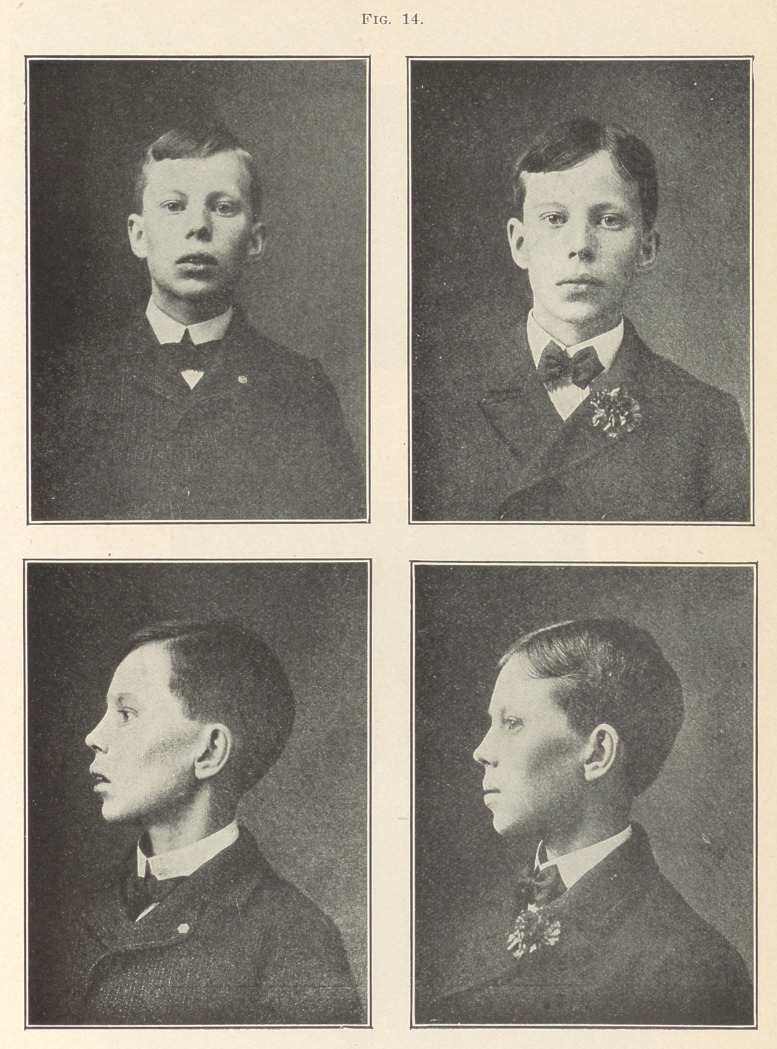


**Fig. 15. f15:**
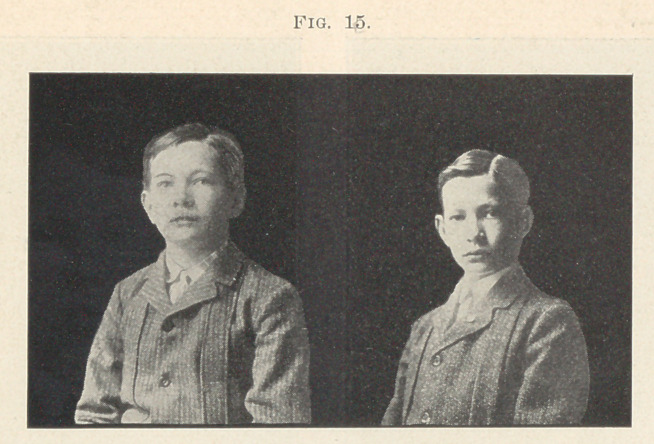


**Fig. 16. f16:**
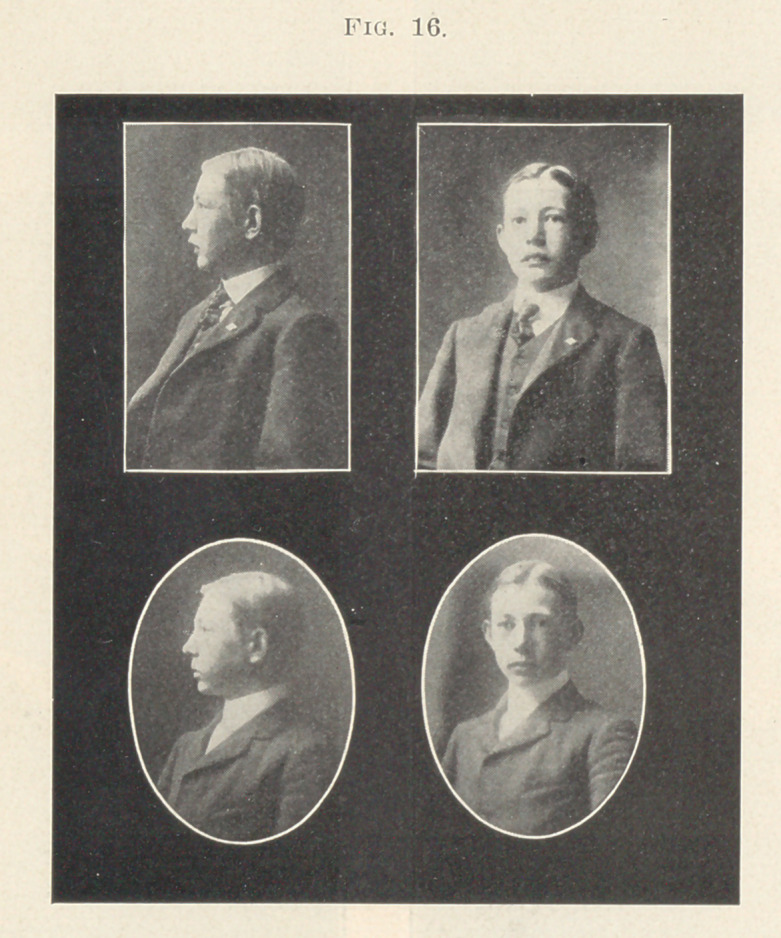


**Fig. 17. f17:**
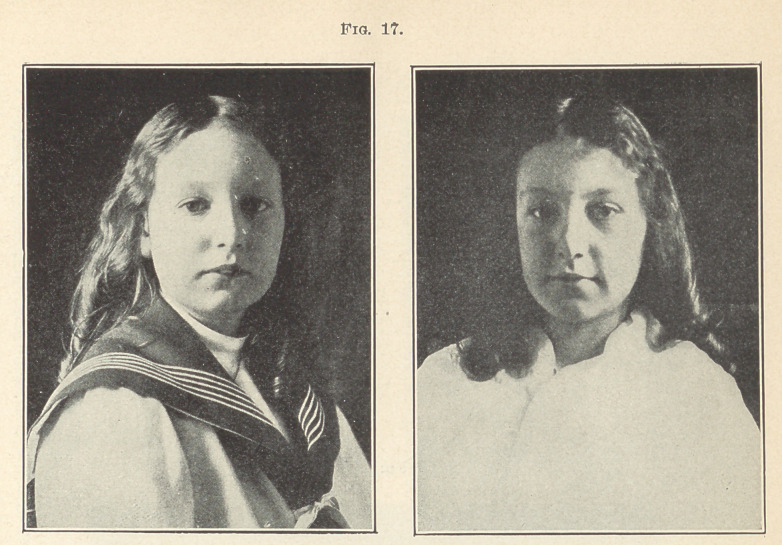


**Fig. 18. f18:**
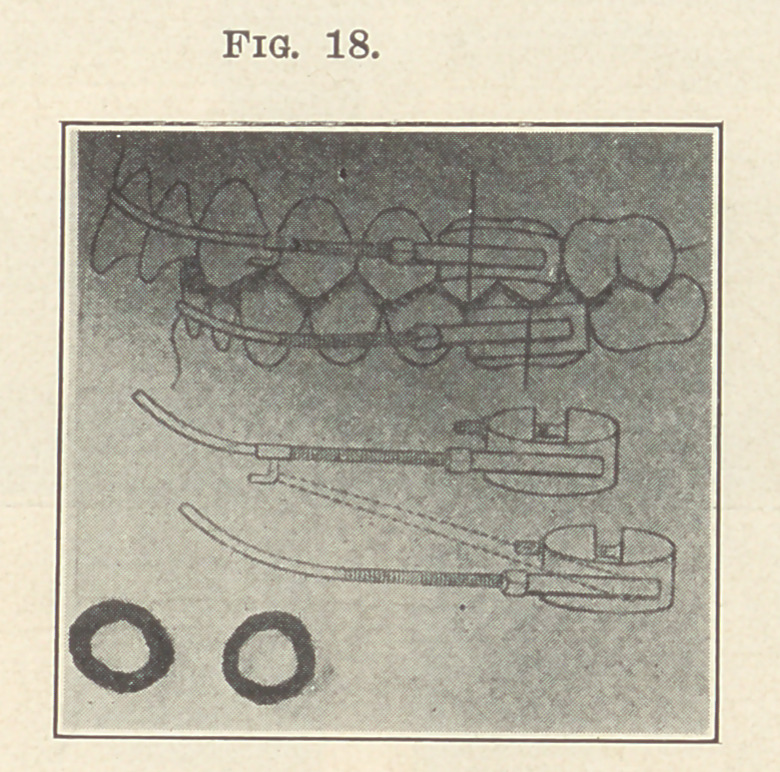


**Fig. 19. f19:**
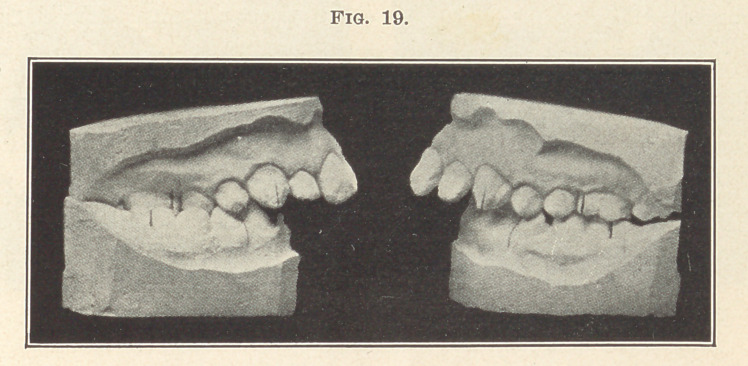


**Fig. 20. f20:**
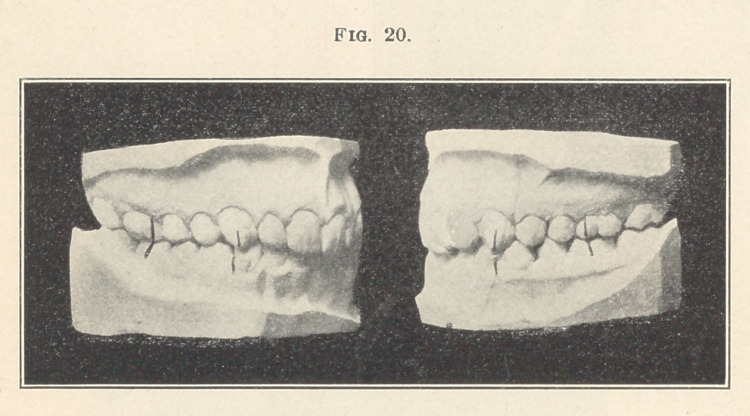


**Fig 21. f21:**
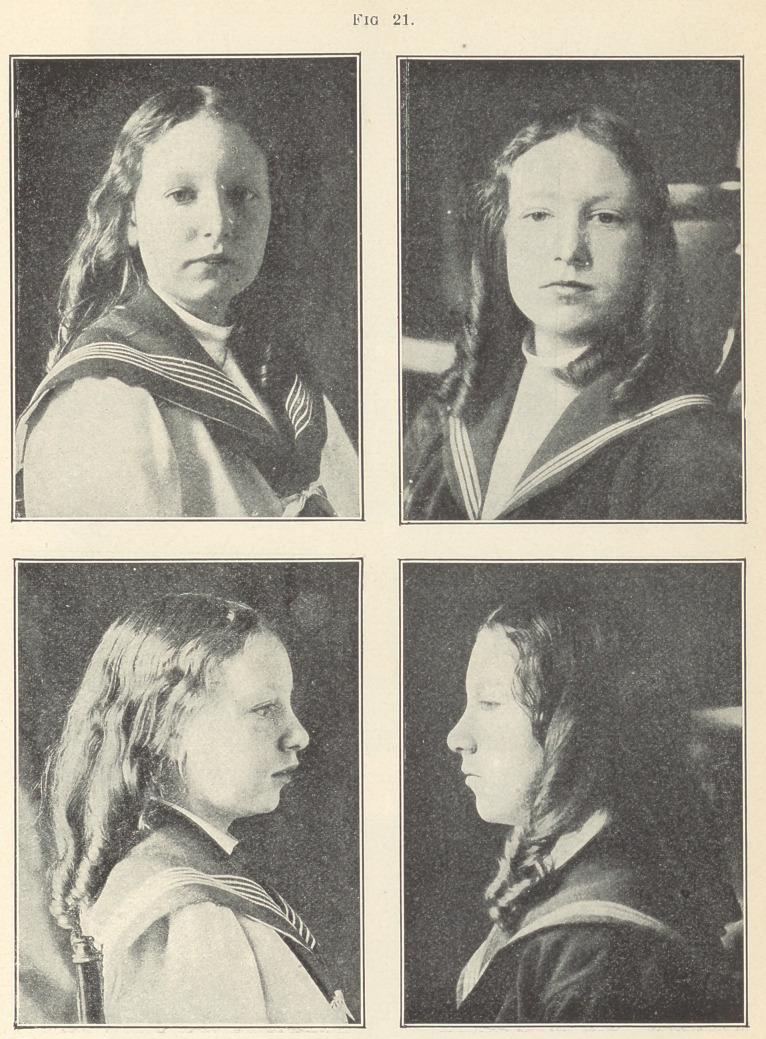


**Fig. 22. f22:**
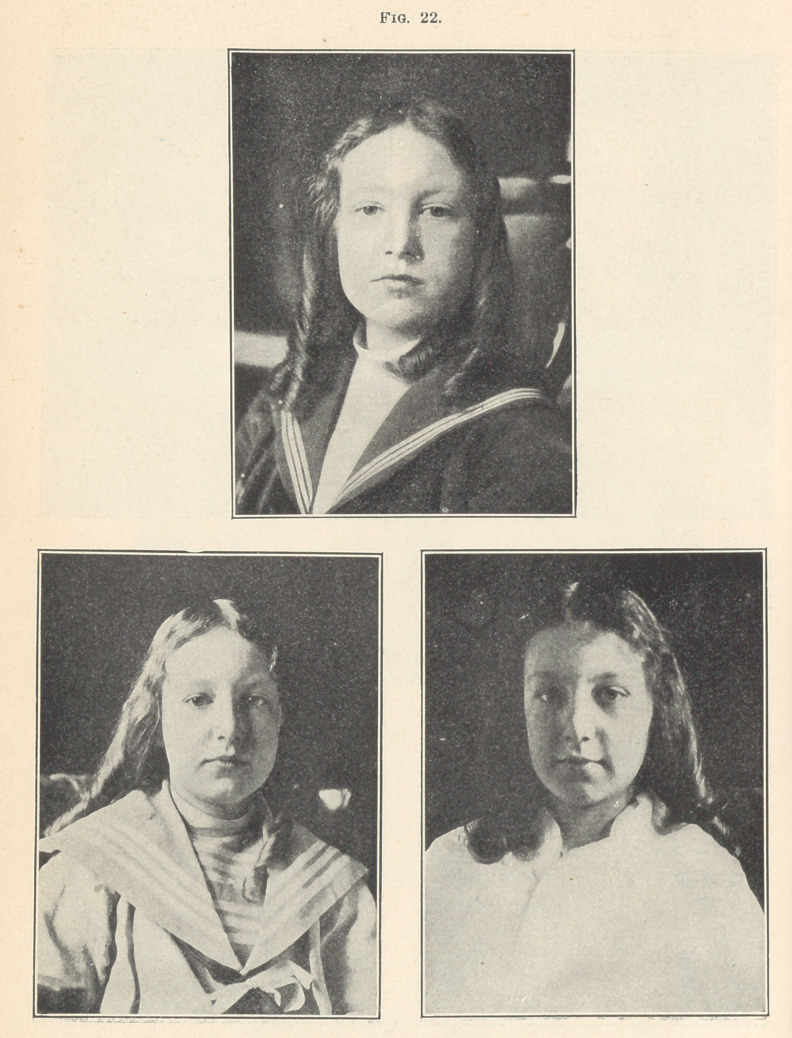


**Fig. 23. f23:**
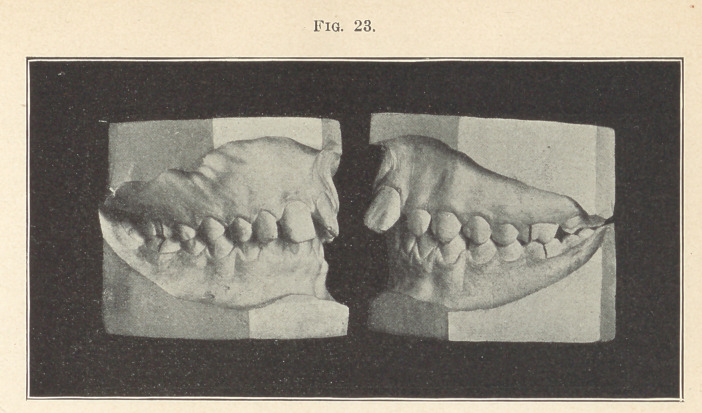


**Fig. 24. f24:**
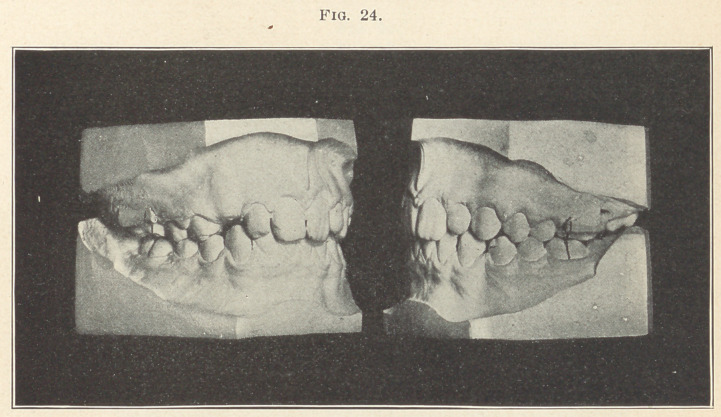


**Fig. 25. f25:**
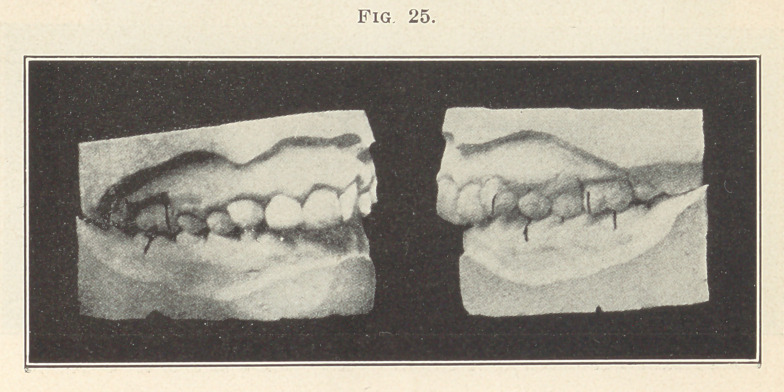


**Fig. 26. f26:**
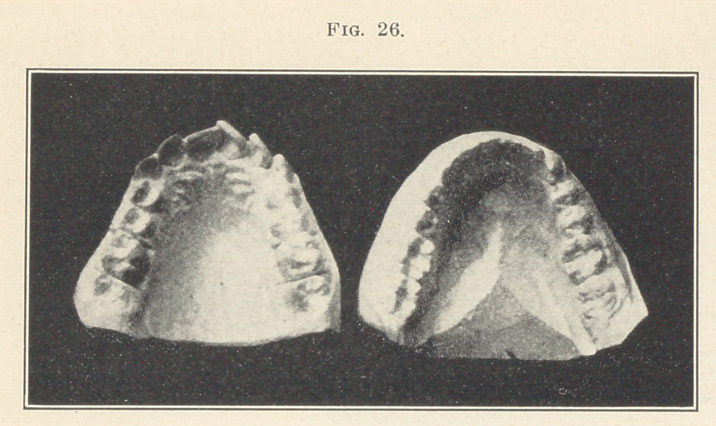


**Fig 27. f27:**
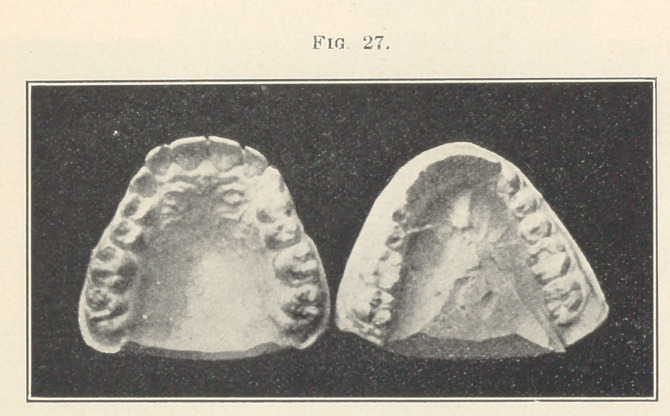


**Fig 28. f28:**
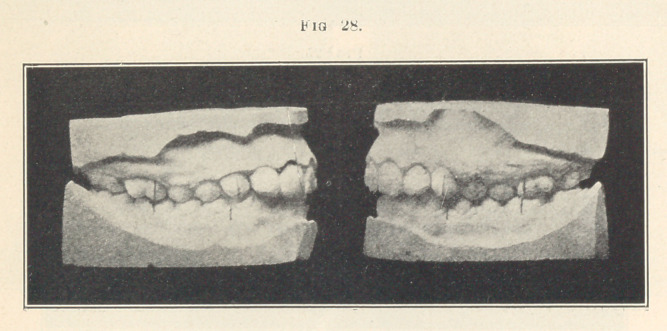


**Fig. 29. f29:**
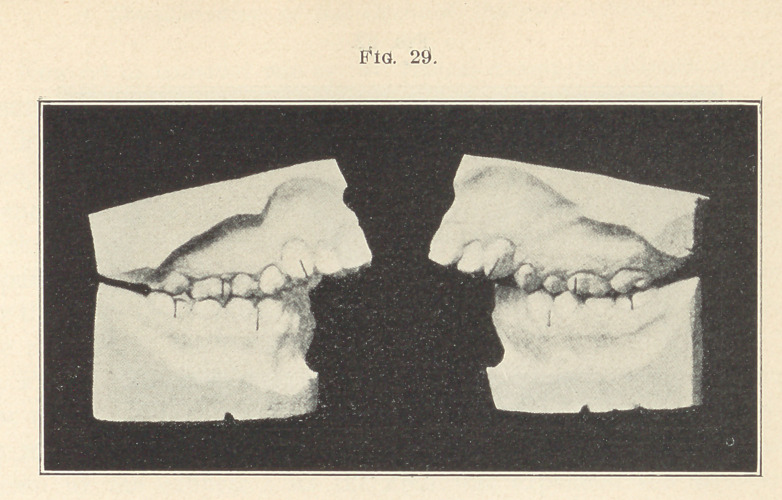


**Fig. 30. f30:**
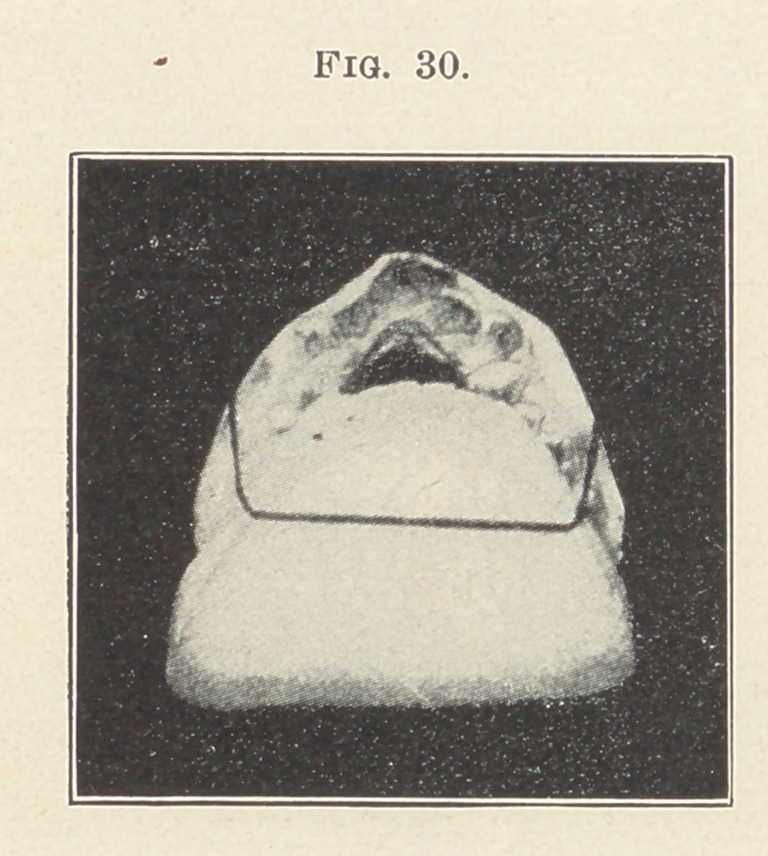


**Fig. 31. f31:**
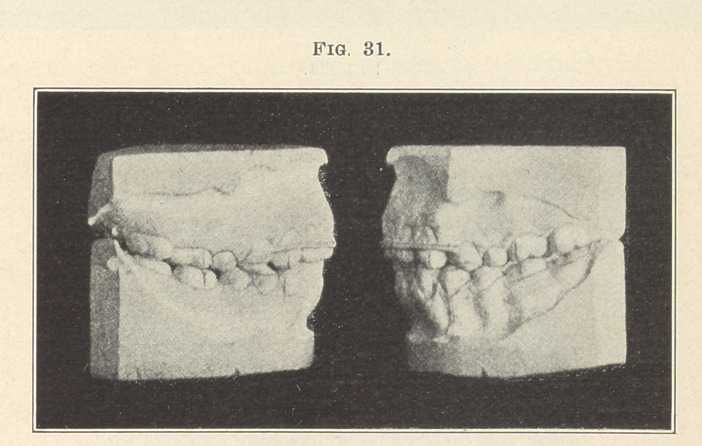


**Fig. 32. f32:**
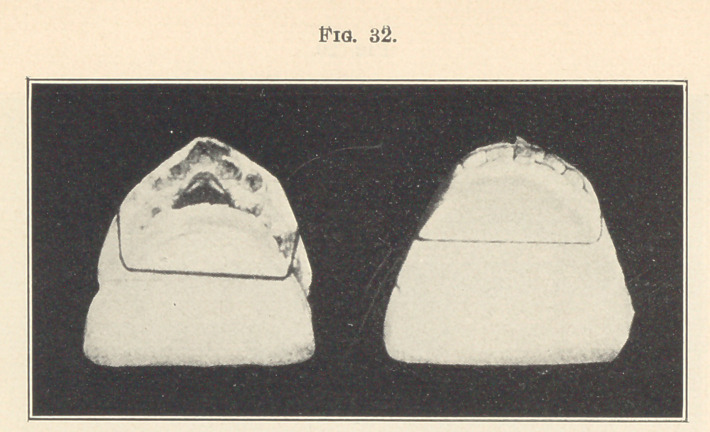


**Fig. 33. f33:**
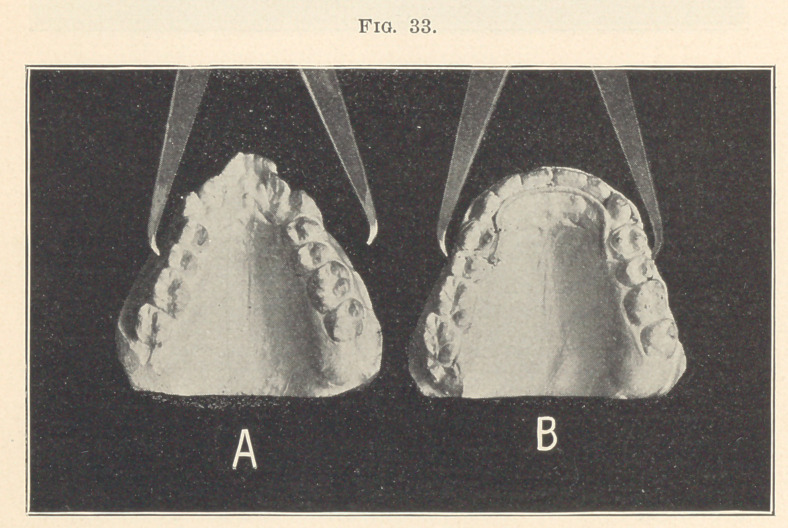


**Fig. 34. f34:**